# Machine learning used for simulation of MitraClip intervention: A proof-of-concept study

**DOI:** 10.3389/fgene.2023.1142446

**Published:** 2023-03-09

**Authors:** Yaghoub Dabiri, Vaikom S. Mahadevan, Julius M. Guccione, Ghassan S. Kassab

**Affiliations:** ^1^ California Medical Innovations Institute, San Diego, CA, United States; ^2^ University of California San Francisco, San Diego, CA, United States

**Keywords:** prediction of MitraClip outcomes machine learning, mitral valve, finite element method, deep learning, XGBoost

## Abstract

**Introduction:** Severe mitral regurgitation (MR) is a mitral valve disease that can lead to lifethreatening complications. MitraClip (MC) therapy is a percutaneous solution for patients who cannot tolerate surgical solutions. In MC therapy, a clip is implanted in the heart to reduce MR. To achieve optimal MC therapy, the cardiologist needs to foresee the outcomes of different scenarios for MC implantation, including the location of the MC. Although finite element (FE) modeling can simulate the outcomes of different MC scenarios, it is not suitable for clinical usage because it requires several hours to complete.

**Methods:** In this paper, we used machine learning (ML) to predict the outcomes of MC therapy in less than 1 s. Two ML algorithms were used: XGBoost, which is a decision tree model, and a feed-forward deep learning (DL) model. The MC location, the geometrical attributes of the models and baseline stress and MR were the features of the ML models, and the predictions were performed for MR and maximum von Mises stress in the leaflets. The parameters of the ML models were determined to achieve the minimum errors obtained by applying the ML models on the validation set.

**Results:** The results for the test set (not used during training) showed relative agreement between ML predictions and ground truth FE predictions. The accuracy of the XGBoost models were better than DL models. Mean absolute percentage error (MAPE) for the XGBoost predictions were 0.115 and 0.231, and the MAPE for DL predictions were 0.154 and 0.310, for MR and stress, respectively.

**Discussion:** The ML models reduced the FE runtime from 6 hours (on average) to less than 1 s. The accuracy of ML models can be increased by increasing the dataset size. The results of this study have important implications for improving the outcomes of MC therapy by providing information about the outcomes of MC implantation in real-time.

## Introduction

The mitral valve (MV) ensures unidirectional blood flow from the left atrium to the left ventricle (LV). Mitral regurgitation (MR) is a pathological condition whereby the MV does not close properly, causing blood reflux to the left atrium during contraction. This disease is the most common cardiac valve disease in the US (Nkomo et al., 2006). Nearly four million people suffer from severe MR, and each year nearly 250,000 new patients are diagnosed (St. Goar et al., 2003; Lloyd-Jones et al., 2010). One treatment option is surgery, but nearly half of patients cannot benefit from this option because of frailty, co-morbidities and/or LV impaired function (St. Goar et al., 2003). Another option that can be more suitable is MitraClip (MC) intervention whereby the cardiologist performs a percutaneous procedure (Fann et al., 2004). The efficacy of this intervention has been studied in clinical trials (Mauri et al., 2013; De Bonis et al., 2015).

Currently, the success of MC intervention is largely based on the cardiologist’s expertise. The intervention itself involves several parameters that can be selected, such as the location and number of MCs, the clip size, and leaflet grasping. To optimize the intervention, the cardiologist should attempt various scenarios with different parameters. Computational models are essential for optimization where virtual placements can be assessed since this is not feasible in patients.

We use physics-based modeling, based on the finite element (FE) method, to create *in silico* models of the MC scenarios. FE modeling can replicate the MR for different parameters in the MC intervention, as reported in previous publications (Dabiri et al., 2019a; Kamakoti et al., 2019; Caballero et al., 2020; Dabiri et al., 2020). However, because FE simulations are time-consuming, they are not suitable for clinical applications because the cardiologist needs the results in real-time for each patient. Furthermore, the optimal MC scenario cannot be directly extracted from FE results because the relation between MC parameters and optimal outcomes of this therapy are complex. As such, the results from FE computations for different scenarios need to be analyzed to determine the best option, which could increase the time required to provide the results from FE simulations.

Machine learning (ML) has been used in cardiovascular mechanics for different applications (Madani et al., 2018; Madani et al., 2019; Gilbert et al., 2020; Quer et al., 2021). We have used ML to predict LV mechanics. In particular, we used decision tree and deep learning (DL) models to simulate important data such as LV stress, pressure and volume, and reduce the FE runtime from several hours to a few seconds (Dabiri et al., 2020; Dabiri et al., 2019b).

To address the limitations of FE simulation of MC therapy, we applied ML-based methods to obtain the outcomes of MR in real-time. For this, we used a dataset of FE models that we created for different MV geometries and MC locations (Dabiri et al., 2021). There were six possible locations for the MC. A decision-tree algorithm, eXtreme Gradient Boosting (XGBoost), as well as feed-forward DL were used to replicate FE results in real-time, whereby the MR and maximum leaflet stress were predicted for each location of the MC.

## Materials and methods

### Data generation

The details of the database generation have been described elsewhere (Dabiri et al., 2021). In brief, our database was created from MV geometries obtained from different resources, including echocardiography patients’ data, morphological data from the literature (Krawczyk-Ożóg et al., 2017), and principal component analysis (PCA). The respective patient images were obtained in accordance with University of California San Francisco Institutional Review Board (number 19-27738). The database provides MR for different MC locations included data from 181 geometries. There were seven scenarios for each geometry, without MC and six locations for the MC. The original dataset was split into training and test sets. The test set was data from eight geometries (56 models), which were not used in the training.

### Decision tree predictions

**FIGURE 1:**
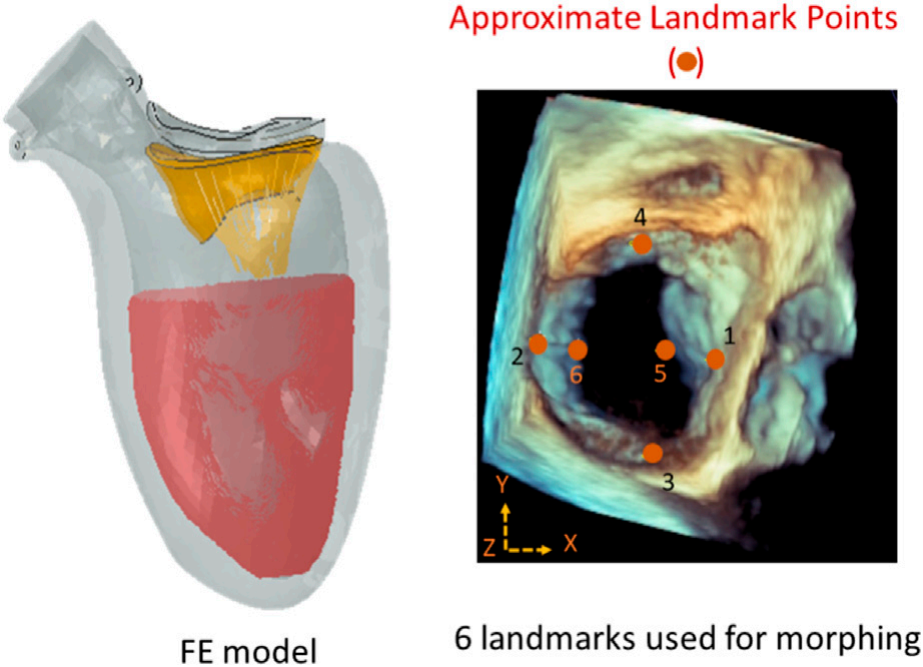
The approximate locations of the landmark points used during morphing (right), and the FE model (left). There were six locations along the leaflet edges where the MC can be placed. The FE model included the mitral valve, the chords, the LV, and blood particles. The features of the ML models were: X_P1_, X_P2_, Y_P3_, Y_P4_, X_P5_, Z_P5_, X_P6_, and Z_P6_ where P1 to P6 are landmark points. The image on the right is from Abaqus (2021, https://www.3ds.com/products-services/simulia/products/abaqus/). Horos (3.3.6, https://horosproject.org/) and ImageJ (1.53e, https://imagej.nih.gov/ij/) were used to create the figure on the right.

We used XGBoost to predict the outcomes of MC therapy, specifically the MR and leaflet stresses, for different scenarios. XGBoost is a decision-tree algorithm that uses a sequence of weak decision trees to make predictions (Dabiri et al., 2019b; Sun et al., 2018). The tree decision-making process of this algorithm has been illustrated in the literature (Chen and Guestrin, 2016). The features of the model included the geometrical parameters of the leaflets and the location of the MC (Figure 1), as well as baseline MR and baseline maximum von Mises leaflet stress. MR or maximum leaflet von Mises stress was the output.

We used a cross-validation technique during training the ML model (Refaeilzadeh et al., 2009; Chicco, 2017). In this technique, the training set was divided into *n* = 3 folds, and each fold was used for validation of the training results, which was based on the other n-1 folds. For this purpose, we used three hyper parameters: learning rate, maximum depth, and number of estimators. For each hyper parameter, several values were used (Table 1). The XGBoost analysis was conducted by the cross-validation algorithm to obtain the hyper parameters with best results. The cross validation was implemented using the GridSearchCV algorithm in sklearn. model_selection library.

**TABLE 1:** Parameters used in the grid search for XGBoost algorithm.

	Learning rate	Maximum depth	Number of estimators
range	0.000001, 0.0001, 0.001, 0.01, 0.1	2, 3, 5, 7	1000, 4000, 10,000
Optimal (MR)	0.01	7	1,000
Optimal (stress)	0.1	3	500

### DL model

A feed-forward DL model was used for MR and stress predictions. The DL model was composed of several hidden layers, the number of which was changed to improve model performance. The first layer, the input layer, corresponds to the attributes of the data, namely, the geometrical specification of landmark points and the location index (1–6) for the MC as well as baseline MR and maximum von Mises leaflet stress. The output layer corresponded to either MR or the maximum leaflet von Mises stress. The number of hidden layers and the number of neurons in each layer were altered based on the validation set error analysis. The L_1_ loss function and Adam optimizer were used to compute the error between predicted and ground truth outputs, and to calculate the updated weights. Advantages of Adam optimizer include computational efficiency, memory requirement, and convenient implementation (Kingma and Ba, 2015). We used the Pytorch library for DL computations (Paszke et al., 2017).

Both XGBoost and DL computations were performed on Google Colaboratory platform, using Python programming language and related libraries. Graphical processing units (GPUs) were used for DL computations. To investigate model performance during training, mean absolute error (MAE) was used, which is the average of the difference between FE and predicted outputs. To assess the accuracy of the predictions, the mean absolute error (MAPE) was utilized. Using the test dataset, the MAPE was computed as follows:

$$MAPE=\frac{1}{n_{samples}}\overset{n_{samples}-1}{\underset{i=0}{\sum }}\frac{\left|y_{i}-{\hat{y}}_{i}\right|}{\max\left(\in ,\left|y_{i}\right|\right)}$$

where ∈ is “an arbitrary small yet strictly positive number”, $n_{samples}$ is number of samples, and $y_{i}$ and ${\hat{y}}_{i}$ are the FE and predicted MR, respectively (Scikit-learnv.1.0.1 documentation).

## Results

The FE models provided the MR and leaflet stresses. The average runtime to complete an FE model was approximately 6 h (Table 2). The FE computations did not converge for all models; when there were FEs with excessive distortion, the model failed to converge. After the FE computations were completed, postprocessing of the results provided different parameters. We focused on MR and leaflet stress as they are important factors for MC therapy.

**TABLE 2:** Runtime (CPU) for FE and ML results.

	MR		Stress	
	XGBoost	DL	XGBoost	DL
ML Training	1,473 s	2,106 s	762 s	3,110
ML Inference	Less than 1s			
FE	In average 6 h			

**FIGURE 2:**
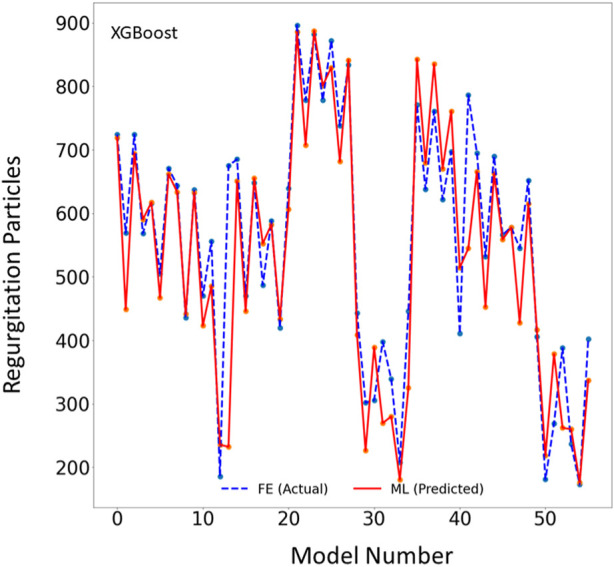
The MR results for the test set obtained from FE (ground truth) and XGboost (predicted). The MR is computed based on the blood particles that leaked into left atrium. Python (3.7, https://www.python.org/) was used to create this figure.

**FIGURE 3:**
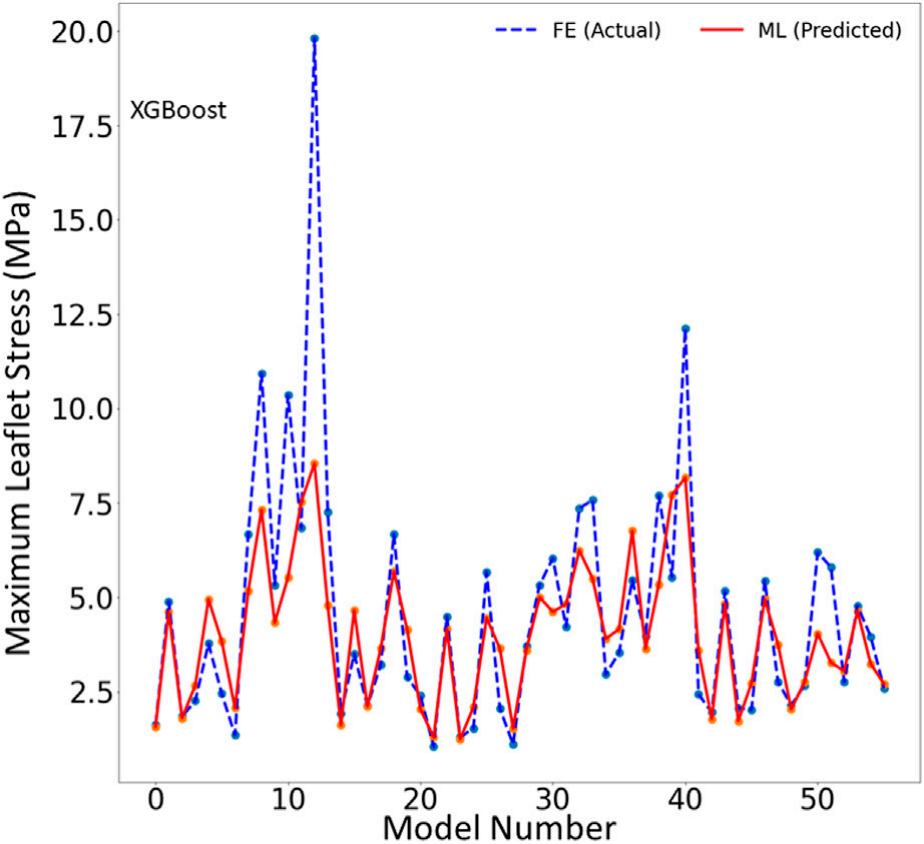
The maximum leaflet von Mises stress for the test set obtained from FE (ground truth) and XGboost (predicted). Python (3.7, https://www.python.org/) was used to create this figure.

**FIGURE 4:**
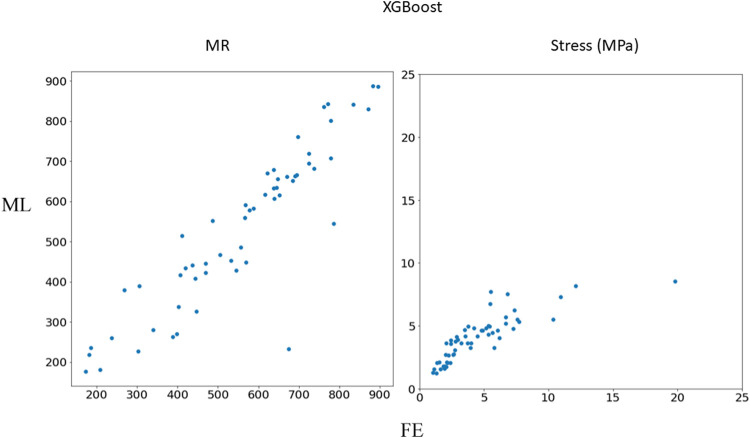
Comparison between FE results and XGBoost predictions. Python (3.7, https://www.python.org/) was used to create this figure.

The XGBoost algorithm predicted the results for MR and maximum leaflet stress (Figures 2–4). A sample tree from XGBoost model for stress predictions is shown in the (Supplementary Figure S1). The accuracy of the predictions was dependent on the hyperparameters. The grid search algorithm provided the optimal hyper parameters (Table 1). For MR the optimal learning rate was 0.01 and maximum depth was 7, and optimal number of estimators was 1,000. For maximum leaflet von Mises stress the optimal learning rate was 0.1 and maximum depth was 3, and optimal number of estimators was 500. The training and inference time for this algorithm are summarized in Table 2, and the MAPE results are summarized in Table 3.

**TABLE 3:** MAPE for different ML models using the testing data.

Model	Quantity	MAPE
XGBoost	MR	0.115
	Stress	0.231
DL	MR	0.154
	Stress	0.310

**FIGURE 5:**
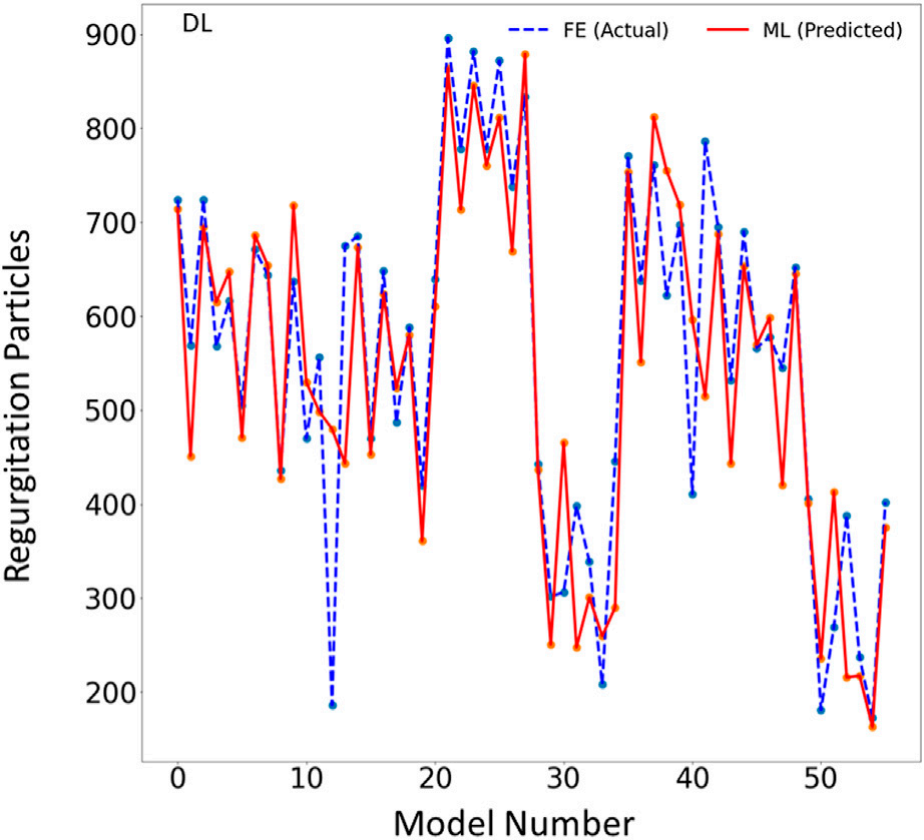
The MR results for the test set obtained from FE (ground truth) and DL (predicted). The MR is computed based on the blood particles that leaked into left atrium. Python (3.7, https://www.python.org/) was used to create this figure.

**FIGURE 6:**
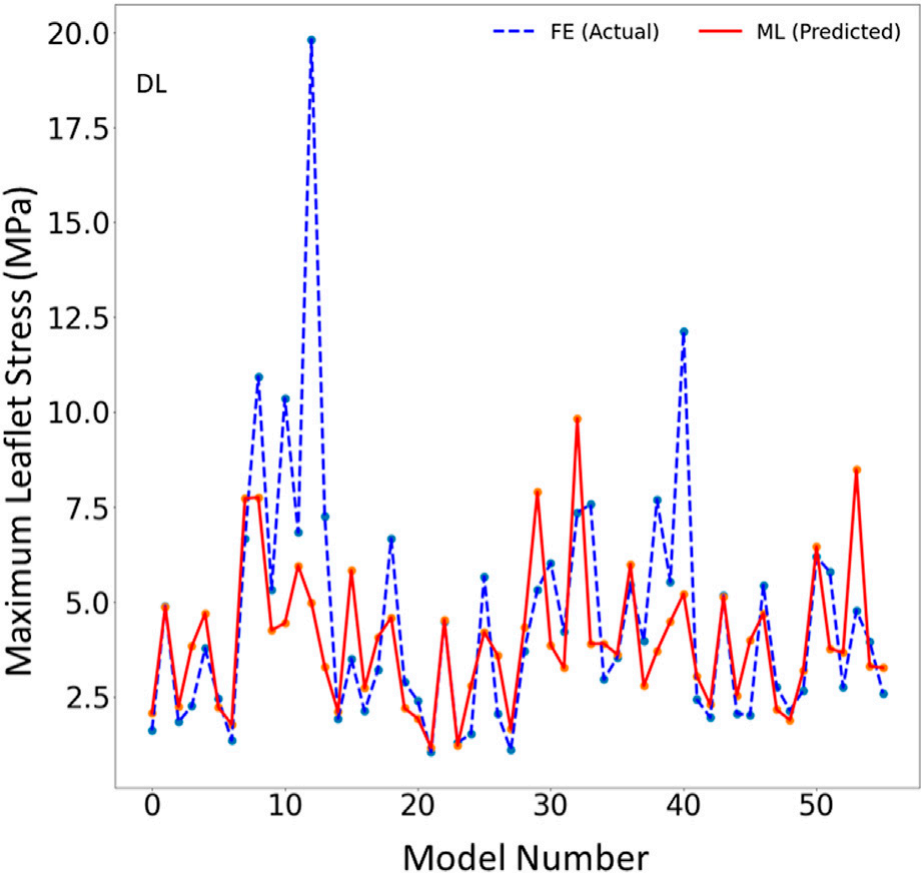
The maximum leaflet von Mises stress for the test set obtained from FE (ground truth) and DL (predicted). Python (3.7, https://www.python.org/) was used to create this figure.

**FIGURE 7:**
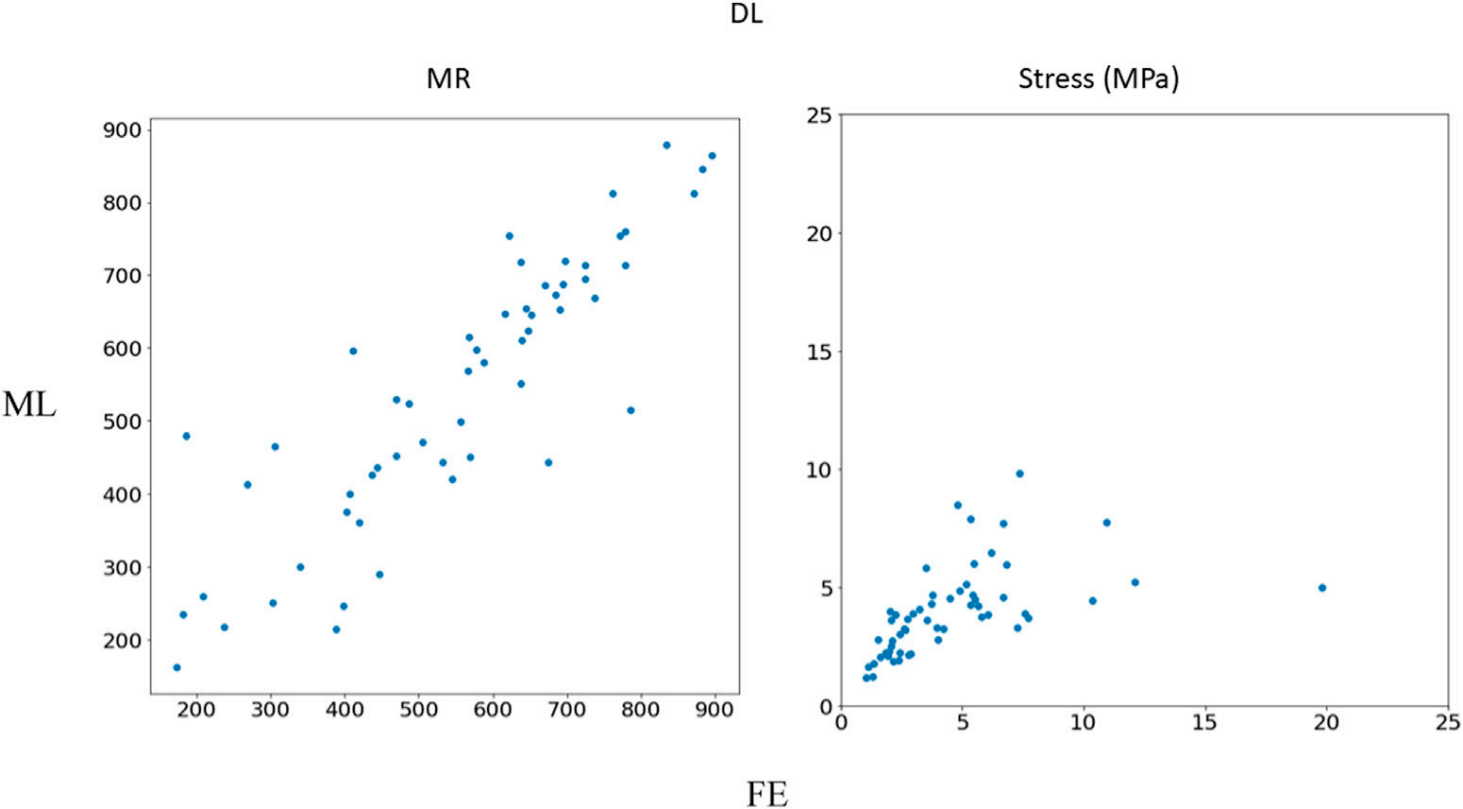
Comparison between FE results and DL predictions. Python (3.7, https://www.python.org/) was used to create this figure.

The DL model also provided the MR and leaflet stresses (Figures 5–7). The DL predictions were sensitive to DL hyperparameters including learning rate, number of epochs, and number of DL layers and neurons. We used 14 layers of neurons. The first layer right after the input layer had 100 neurons, the two next layers had 200 neurons, and the other layers except for the output layer (one output) had 300 neurons. The runtime and MAPE are summarized in Tables 2, 3, respectively.

**FIGURE 8:**
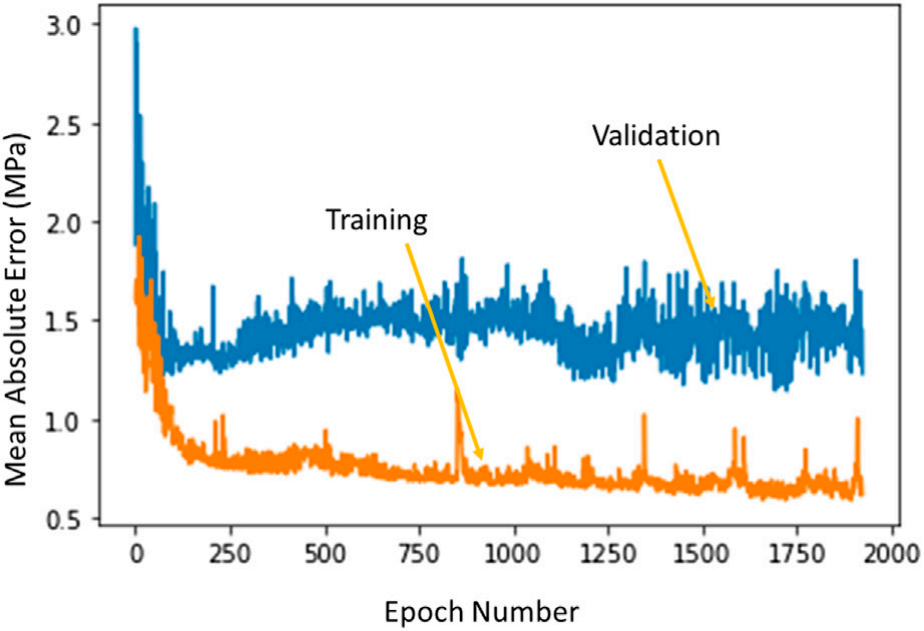
The training error and test error change with number of epochs in DL algorithm. The XGBoost errors have a similar behavior. Python (3.7, https://www.python.org/) was used to create this figure.

The ML model errors had different characteristics for training and validation sets. The training errors continued to decrease as the number of estimators (XGBoost), or number of epochs (DL) increased. However, the validation set error had a saddle point after which the error started to increase. The XGBoost model hyperparameters were based on the errors obtained from different folds of the validation sets. The DL optimal hyperparameters were selected based on the error for the validation set whereby the hyperparameters that provided the minimum validation error were selected for the DL model (Figure 8).

According to the XGBoost analysis, different features used for ML predictions had different levels of importance (Table 4). For MR predictions, baseline MR had the highest level of importance whereas for stress predictions, baseline stress was the most important feature. The importance of geometrical features for MR predictions were different, implying different levels of effect on MR. Additionally, different geometrical attributes had different levels of importance for stress predictions. Moreover, for MR and stress outcomes the importance of each geometrical feature was different.

**TABLE 4:** Feature importance for MR and stress predictions.

	Importance	
	MR	Stress
Feature		
Clip location	0.050	0.027
X_P1_	0.009	0.012
X_P2_	0.075	0.023
Y_P3_	0.022	0.023
Y_P4_	0.022	0.004
X_P5_	0.025	0.028
Z_P5_	0.037	0.059
X_P6_	0.009	0.022
Z_P6_	0.014	0.045
Baseline stress	0.009	0.717
Baseline MR	0.727	0.041

## Discussion

In this study, we used ML to predict the outcomes of MC therapy in less than 1 s, in contrast to FE computations, which need 6 h on average. We focused on MR and maximum leaflet stress, as they (especially MR) are critical for successful MC therapy. Although we used a relatively small dataset (1267 FE models), the ML models provided results much faster than FE and with relatively reasonable accuracy (Figures 2–7; Tables 2, 3). To the best of our knowledge, our study is the first one that uses ML for prediction of MC outcomes.

One of the two ML models we studied, the XGBoost algorithm, predicted the MR and leaflet stress with relatively reasonable accuracy (Figures 2–4). The prediction accuracy depends on several factors. The hyperparameters (including number of estimators), learning rate and maximum tree depth were altered by the cross-validation algorithm, which finds the optimal model. These parameters can change the accuracy of the predictions to some extent. Another important factor in achieving accurate ML predictions is the data size. The XGBoost training was based on only 1267 FE models (datapoints). This relatively small dataset could explain why the XGBoost algorithm failed to predict results for some cases with relatively high accuracy (Figures 2–4).

The DL model training was also based on 1267 FE models (datapoints). We tried different sets of parameters to improve the DL results, specifically altering the number of layers and number of neurons in each layer, as well as the learning rate. The accuracy of the DL model was noticeably altered by changing these parameters. However, as with XGBoost, DL failed to predict the results for some cases with relatively high accuracy (Figures 5–7). The errors in the DL predictions can be explained by the optimal hyperparameters and the relatively small dataset.

Analysis of feature importance shows that the baseline MR and stress were the most important in predicting the outcomes of MC intervention (Table 4). However, because other specifications of the ML models were the same, the baseline MR and stress are influenced only by the MV geometry. We used the geometrical landmarks utilized during the morphing process. If the MV geometry is encoded in other dimensions, such as the principal components (Liang et al., 2018), the importance of baseline MR and stress could be affected. Consequently, some features in the encoded dimensions could become the most important features.

Results showed that XGBoost provided higher accuracy than DL (Table 3). However, this should not be generalized. There could be a structure of DL models that provide results with comparable accuracy with XGBoost. Since DL models are usually large models, they are more suitable for large datasets. Given the relatively small dataset in this study, we expected that XGBoost can better predict the FE results. Moreover, the accuracy for MR was better that stress (Table 3). This can be related to numerical errors in FE data. We used maximum von Mises stresses in the leaflets. This FE result can be affected by element distortions in such a way that some maximum stress data act like noise in the dataset and affect the ML models performance.

The clinical benefits of using ML over FE to predict MC outcomes are considerable. First, FE modeling is time-consuming and therefore impractical for predicting the possible undesired consequences of MC therapy in a given patient. ML provided the results in less than 1 s, which addresses the need for rapid results required in the clinic. Second, FE calculations are much more expensive than ML analysis, in terms of both required hardware and software. However, FE convergence is not guaranteed. If the geometry is not accurate or is not well-defined, there is a chance that FE models do not converge. On the other hand, ML analysis is less sensitive to these factors. For example, if the image provides the six landmark points, the ML can provide the results. Moreover, if the data provides just a few of the six landmark points, statistical analysis can be used to approximate the missing landmarks. Importantly, modeling the relation between MC outcomes and image data requires ML algorithms that can extract complex relations between attributes (MV geometrical features and MitraClip specifications) and outcomes (MR and leaflet stress).

## Limitations and future directions

One limitation of this work is the relatively small size of the dataset used, which included only 1,267 datapoints. This limitation is particularly important for the ML predictions. As more data becomes available, we will re-train our ML algorithms. For example, the points in Figures 3, 6 which have higher errors, also have higher stress values as compared to other points. If the number of datapoints is increased, the ML model parameters will be adapted for these cases so that the respective error decreases. With a larger dataset, the overfitting problem for all datapoints also improves, leading to better overall predictions. Nevertheless, this paper, which describes a proof-of-concept study, takes a step forward for using ML to provide MC therapy outcomes for clinical use.

Other ML algorithms such as random forest, support vector machines (SVM) and recurrent neural networks can be used to predict FE results. Our computations (not shown in this paper) with random forest and SVM did not show better performance as compared to XGBoost and DL. In our study, we used XGBoost and feed-forward DL. XGBoost can be used for small datasets, it is computationally efficient and its advantages over other decision tree models include runtime, sensitivity to outliers, flexibility in processing data, and performance (Chen and Guestrin, 2016; Sun et al., 2018). We previously showed that for LV mechanics, DL can provide results in higher accuracy for a relatively small dataset (Dabiri et al., 2020). The other model used in the current study, feed-forward DL, was chosen for computational efficiency because recurrent neural networks usually require more computations for similar tasks. The DL performance was not as good as XGBoost (Figures 4, 7; Table 3). DL model complexity can be higher than that of XGBoost if number of layers and neurons increases. Therefore, XGBoost may be preferred for its computational efficiency and lower generalization errors. This condition is more important for cases where model complexity is too high with respect to the dataset size.

It should be noted that we used different number of hidden layers, neurons, and hyper parameters to achieve best results in terms of DL predictions error. The results shown in this paper were obtained from the structure with lowest error. We used a try and error approach, but there are algorithms to find optimal DL structures, such as genetic algorithms which can be implemented as a future direction (Stathakis, 2009).

Minimal MV area is another important consideration for MC intervention. In this paper, we presented mitral regurgitation and leaflet stresses. We did not present results for MV area for two reasons: 1) MV area and regurgitation are related; and 2) Our focus was MV regurgitation. In other words, the results presented for MV regurgitation and stress can be repeated for MV area.

In summary, severe MR is a disease that is fatal if untreated, and has high burden on the healthcare system. MC implantation can be an effective option for patients who cannot tolerate surgery, but because the procedure currently relies on the cardiologist’s intuition, it could lead to adverse outcomes. We used ML to predict the outcomes of MC implantation in near real-time (less than 1 s), which has important ramifications in clinical practice.

## Data Availability

The data analyzed in this study is subject to the following licenses/restrictions: The datasets generated and/or analyzed during the current study are not publicly available due to limitations in data access policy in corresponding institutes but are available from the corresponding author on reasonable request. Requests to access these datasets should be directed to gkassab@calmi2.org.
