# Supplementary material for: Machine learning used for simulation of MitraClip intervention: A proof-of-concept study
Source: Front Genet. 2023 Mar 9;14:1142446. doi: 10.3389/fgene.2023.1142446 (PMC10033889; doi:10.3389/fgene.2023.1142446)
Supplement: Supplementary file 1 [file Image1.pdf]

# Supplementary Materials

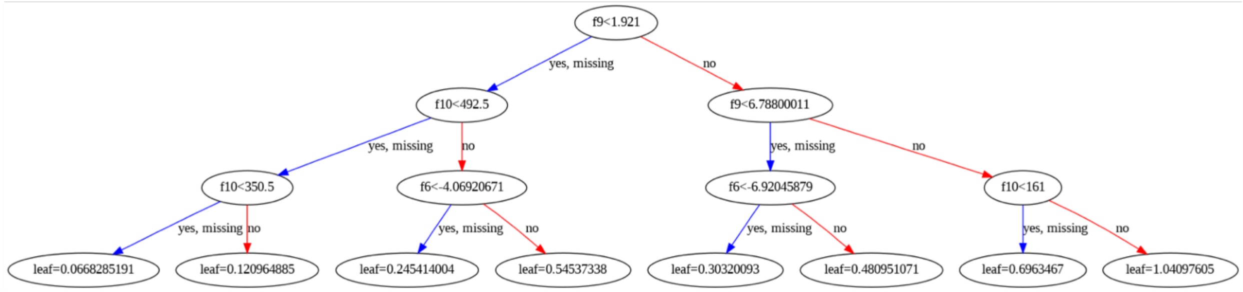


Fig. S1: A sample tree in the XGBoost model for stress predictions. This is the first tree in the model.
